# Lysosomal function in macromolecular homeostasis and bioenergetics in Parkinson's disease

**DOI:** 10.1186/1750-1326-5-14

**Published:** 2010-04-13

**Authors:** Lonnie Schneider, Jianhua Zhang

**Affiliations:** 1 Department of Pathology, University of Alabama at Birmingham, Birmingham, AL35294, USA

## Abstract

The pathological changes occurring in Parkinson's and several other neurodegenerative diseases are complex and poorly understood, but all clearly involve protein aggregation. Also frequently appearing in neurodegeneration is mitochondrial dysfunction which may precede, coincide or follow protein aggregation. These observations led to the concept that protein aggregation and mitochondrial dysfunction either arise from the same etiological factors or are interactive. Understanding the mechanisms and regulation of processes that lead to protein aggregation or mitochondrial dysfunction may therefore contribute to the design of better therapeutics. Clearance of protein aggregates and dysfunctional organelles is dependent on macroautophagy which is the process through which aged or damaged proteins and organelles are first degraded by the lysosome and then recycled. The macroautophagy-lysosomal pathway is essential for maintaining protein and energy homeostasis. Not surprisingly, failure of the lysosomal system has been implicated in diseases that have features of protein aggregation and mitochondrial dysfunction. This review summarizes 3 major topics: 1) the current understanding of Parkinson's disease pathogenesis in terms of accumulation of damaged proteins and reduction of cellular bioenergetics; 2) evolving insights into lysosomal function and biogenesis and the accumulating evidence that lysosomal dysfunction may cause or exacerbate Parkinsonian pathology and finally 3) the possibility that enhancing lysosomal function may provide a disease modifying therapy.

## Introduction

A long-term objective in the study of neurodegenerative diseases is to identify the mechanisms of how cells cope with damage to protein and organelles. Ultimately, this should aid in the design of novel and effective treatment strategies. The failure to clear effectively aged and damaged proteins and organelles is implicated in aging and several neurodegenerative diseases. The lysosome is the high capacity organelle responsible for the normal process of protein and organellar degradation [1-4]. The appearance of aggregated proteins in the cells associated with neurodegenerative diseases is common and suggests a dysfunction in the normal protein degradation pathways including the lysosome [5,6].

α-synuclein accumulation is commonly associated with Parkinson's disease. Mutations in this protein or gene amplification cause a small subset of Parkinson's disease cases, with Lewy body formation, neurodegeneration and often an associated dementia [7,8]. Further, in many sporadic Parkinson's patients who do not carry mutations or upregulation of expression, α-synuclein aggregation is also widespread [9,10], implicating a dysfunction of its degradation in neurons. Further, supporting a central role of α-synuclein in disease pathogenesis, its reduction in experimental models is neuroprotective [11-15]. These observations suggest the therapeutic strategy of enhancing lysosomal function in treatment of Parkinson's and other neurodegenerative diseases. This review will summarize our current understanding of Parkinson's disease with respect to α-synuclein aggregation and mitochondrial dysfunction, examine how lysosomal function is regulated and review the evidence supporting the hypothesis that enhancing lysosomal function is neuroprotective.

## 1. Parkinson's Disease

Parkinson's disease is the second most common neurodegenerative disease and the most common movement disorder, affecting 1% of the population above the age of 60. Its clinical features include tremor, muscle rigidity, bradykinesia and postural instability. These debilitating symptoms are attributed primarily to degeneration of substantia nigra dopaminergic neurons. Parkinson's disease is associated with both genetic and non-genetic contributing factors, with aging as the most prominent risk factor. Both protein aggregation and mitochondrial dysfunction feature prominently in Parkinson's disease [16]. These two disease features and their inter-connection are summarized below.

### 1a. α-synuclein aggregation in Parkinson's disease

Lewy body formation with α-synuclein accumulation is a prototypical pathological feature in Parkinson's and other Lewy body diseases. α-synuclein is a 140 amino acid protein which has a propensity to associate with membranes [17,18]. Its interaction with membranes of different composition can change both the membrane structure and the tendency of α-synuclein to form fibrils [19-25]. Not surprisingly, α-synuclein is enriched at synaptic termini in neurons [26,27], where it is thought to modulate synaptic vesicle release and neuronal fatty acid composition [26-28]. α-synuclein association with the synapse may be regulated by synaptic activities [29] and is increased during learning [30].

Patients with α-synuclein A53T, A30P, or E46K mutations develop typical Parkinson's disease, with Lewy body formation, neurodegeneration and often an associated dementia [7,8]. These mutations in α-synuclein alter its dynamic interactions with membranes [31]. Triplication of the wildtype α-synuclein gene has also been shown to cause Parkinson's disease [8], lending to the idea that increased levels of α-synuclein protein may play a crucial role in the development of idiopathic Parkinson's disease. Further support for this notion is the finding that α-synuclein overexpression leads to its aggregation and/or neurotoxicity in animal models [32-34].

The intracellular concentrations of α-synuclein protein are regulated at mRNA transcription, protein synthesis and targeting, or protein degradation levels. Blockade of α-synuclein production by ribozyme or siRNA could reduce α-synuclein production [11-15], but may not be effective to clear already formed toxic species. Recent studies on α-synuclein transcription regulation may provide clues on how to turn off α-synuclein production [35-37]. Conditionally turning off α-synuclein production after inclusion body formation in a mouse model attenuated progression but did not reverse α-synuclein over-production-induced deficits [38]. One therapeutic strategy is immunization with antibodies [39] to reduce α-synuclein proteins, but this is limited by the requirement for exposure of specific epitopes on the protein and access across the blood-brain barrier.

Furthermore, in >90% of Parkinson's disease cases, and almost all Dementia with Lewy bodies and Lewy body variants of Alzheimer's disease cases, the α-synuclein gene is not mutated nor is α-synuclein mRNA overexpressed [40,41]. Nonetheless, α-synuclein aggregates are invariably present as a main component of the Lewy body and levels of this protein are increased in both Triton X-100 soluble and insoluble fractions of whole cell extracts [9,10]. These observations suggest that defective α-synuclein protein degradation is a more important pathogenic factor than α-synuclein overproduction in these cases. In addition to accumulation of protein aggregates, mitochondrial deficits have also been observed to occur in Parkinson's and other neurodegenerative diseases.

### 1b. Mitochondrial defects in Parkinson's disease

Parkinson's disease brains exhibit a reduction in mitochondrial complex I activity, which is both the rate-limiting step for mitochondrial respiratory chain activity, and an important site for generation of reactive oxygen species [42,43]. Reduction of complex I activity may lead to accumulation of reactive oxygen species, which can further induce mitochondrial permeability transition, ATP depletion, and damage of DNA, lipids and proteins. Similar mitochondrial defects are also seen in the Parkinson's disease patients' cortex, in addition to the substantia nigra, suggesting that the reduced mitochondrial activity is not solely due to the extensive (can be as high as 80%) dopaminergic neuron loss in the substantia nigra [43-45]. Mitochondrial complex II, III and V proteins are also reduced in sporadic Parkinson's disease brains [46].

Somatic mtDNA mutations and mitochondrial dysfunction are increased with aging and have been found in Parkinson's and other neurodegenerative diseases [45,47-50]. In addition, recently studies have indicated that mitochondrial dynamics may be defective in Parkinson's disease, including abnormalities in axonal transport, mitochondrial fission and fusion. Axonal degeneration and associated mitochondrial defects occur in a number of neurodegenerative diseases [51-53], particularly because of the long distances the axons in affected neurons traverse. Neurodegeneration may be initiated in the axons or even the synapse, and subsequently extended to the cell body [52,54].

Mitochondrial dysfunction may be induced by environmental toxins that could possibly contribute to Parkinson "like" diseases. For example, 1-methyl 4-phenyl 1,2,3,6-tetrahydropyridine (MPTP) was originally discovered as a byproduct of illegal heroin production, and was shown to cause a Parkinsonian syndrome in drug users [55-58]. MPTP is the neurotoxin that crosses the blood-brain barrier in a matter of seconds, whereby it is rapidly converted to 1-methyl-4-phenylpyridinium (MPP+) by endogenous monoamine oxidase B. MPP+ is able to selectively enter dopaminergic neurons of the substantia nigra based on its size and charge facilitating entry through the dopamine transporter. Upon entering dopaminergic neurons, MPP+ accumulates in mitochondria where it binds to and inhibits complex I, causing ATP depletion, and increasing reactive oxygen species [59,60]. Pesticides rotenone and paraquat that directly and indirectly target to the mitochondria have also been implicated in animal models of Parkinsonism [59,61-64].

These data suggest that mitochondrial dysfunction is not simply an inconsequential phenotype associated with Parkinson's and other neurodegenerative diseases, but in fact can be an initiating factor for pathogenesis. In support of this idea, if mitochondria isolated from Parkinson's disease patients are transferred into mitochondria-depleted neuroblastoma cells, inclusion bodies are formed [65,66]. Decreased mitochondrial biogenesis in MitoPark mice, with deficient mitochondrial transcription factor (Tfam) in midbrain dopaminergic neurons, led to adult onset and progressive Parkinsonism, and associated intraneuronal inclusions [67]. Furthermore, genes identified in rare inherited forms of Parkinson's disease are involved in regulating mitochondrial function [68].

### 1c. Relationship between α-synuclein accumulation and mitochondrial dysfunction in Parkinson's disease and experimental models

Both α-synuclein aggregation and mitochondrial dysfunction feature prominently in Parkinson's disease [16]. Whether these two features are interactive has inspired interesting studies in Parkinson's disease brains and experimental models. α-synuclein interacts with the mitochondrial inner membrane [69,70], and accumulates at greater levels in the mitochondria of substantia nigra and striatal neurons in human Parkinson's disease brains compared to normal brains [70]. In vitro, α-synuclein is targeted to mitochondria via an N-terminal cryptic sequence [70], and decreases complex I activity and the production of reactive oxygen species [70-72]. α-synuclein can also bind to mitochondrial complex IV, and is a possible further mechanism leading to mitochondrial dysfunction [73].

Sub-chronic administration of mitochondrial toxin MPTP has been shown to transiently induce α-synuclein accumulation, followed by extensive cell death, precluding further α-synuclein accumulation [74]. Chronic administration of MPTP together with probenecid leads to the accumulation of Lewy body-like structures that are α-synuclein positive [75-77]. Probenecid is a uricosuric agent that decreases renal excretion of MPTP, thereby sustaining a higher level of MPTP in the brain compared to MPTP alone without probenecid [78,79]. Furthermore, α-synuclein knockout mice are resistant to MPTP-induced dopaminergic neuron death [11-14]. Reduction of α-synuclein by siRNA or ribozyme approaches is neuroprotective in vitro and in mitochondrial neurotoxin-based animal models [15,80]. Transgenic mice overexpressing human α-synuclein exhibit increased pathology in response to MPTP treatment [72,81,82]. These studies suggest that a lysosomal dysfunction that is presumably inadequate to remove accumulated α-synuclein may mediate mitochondrial dysfunction-caused neurodegeneration. A better understanding of the underlying mechanisms is critical to our understanding of Parkinson's disease pathogenesis.

## 2. Lysosomes and their role in neurodegenerative diseases

Lysosomes are acidic intracellular compartments that provide inherent protein-, lipid-, nucleic acid-and pathogen-degrading activities for the cell. They receive their substrates from endocytosis, phagocytosis and autophagy, and are active participants in bulk degradation of metabolic waste products [1]. For a long time, lysosomal enzyme functions were considered as merely downstream events that passively degrade materials that are transported to the organelle. Accumulating evidence however, has indicated that lysosomal activities are rate-limiting, induced under stress, and the major force in clearance of damaged or aggregated proteins [2,5,6,83,84]. In addition, they may even be vital in coordinating intracellular signaling and metabolic activities. Here we will evaluate relevant information regarding lysosomal functions and biogenesis, pertinent to neurodegenerative diseases. We will review current knowledge regarding regulation of normal lysosomal activities, and then summarize the involvement of lysosomal dysfunction in Parkinson's disease.

### 2a. Regulation of normal lysosomal activities

Nearly 100 lysosomal proteins are involved in the structure and activities of the organelle [85]. Almost all of these lysosomal proteins are indispensable for normal physiology in mammals, i.e., almost all have unique and non-redundant roles in the lysosome [5]. Some of the lysosomal enzymes are regulated at the transcriptional level by growth factors [86]. To ensure their coordinated function, the expression of many of the lysosomal proteins is regulated by common mechanisms [87].

**Figure 1:**
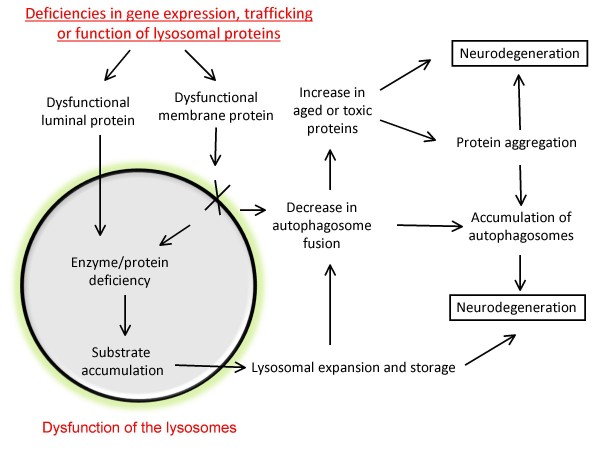
**Lysosomal deficiencies that may lead to neurodegeneration**. Functions of lysosomes are regulated at multiple levels, including coordinated transcriptional regulation of lysosomal genes, trafficking of lysosomal proteins to the lysosomes, and proper function of the lysosomal membrane proteins and luminal acid hydrolases. Deficiencies of any of these processes may lead to deficiencies of reduced lysosomal degradation of aged or toxic proteins. The lysosomal substrate accumulation may in turn result in lysosomal expansion and storage and further disruption of its activities. Accumulation of aged, toxic or aggregated proteins and organelles, and accumulation of autophagosomes may lead to eventual neurodegeneration.

One recent landmark work by Sardiello et al has provided new insights into how expression of lysosomal proteins is regulated. Using a bioinformatics approach, a large proportion (68 out of 96 genes) of all lysosomal genes examined contain a palindromic 10 base pair sequence (GTCACGTGAC) in the promoter regions that is a consensus binding site for a basic helix-loop-helix (bHLH) transcription factor EB (TFEB) [87]. Conversely, an unbiased microarray analyses have shown that the 291 genes upregulated by TFEB are enriched for lysosomal genes by a stringent conservative criteria. Additional non-lysosomal TFEB-regulated genes play important roles in transporting enzymes and substrates to the lysosome. With TFEB upregulation, the overall lysosomal volumes are also increased, as measured by lysosomal membrane markers as well as ultra-structural studies. Coordinated regulation of lysosomal and vesicle transport protein levels may serve to ensure maximum efficiency of the system in times of stress, as well as highlight the cooperative nature of the acid hydrolases during macromolecular degradation. Furthermore, TFEB genetic polymorphisms may contribute to alterations of its function and predisposition to neurodegenerative diseases via alterations of transcription of lysosomal proteins (Figure 1).

In addition to gene expression, lysosomal activities are also regulated when proteins are targeted to the organelle. To ensure proper targeting, multiple pathways select and deliver lysosomal proteins to their final destinations. Trafficking of the more than 50 known lysosomal acid hydrolases is largely dependent on a mannose-6-phosphate receptor (M6Pr)-mediated mechanism, although processes independent of mannose-6-phosphate receptor also exist. The mannose-6-phosphate receptor (M6Pr)-dependent pathway is the most common pathway for trafficking of the acid hydrolases in the lysosomal lumen. Typically, the lysosomal acid hydrolases are synthesized with an N-terminal signal peptide that is recognized by the signal recognition particle. The signal recognition particle enables the proteins to be translocated into the endoplasmic reticulum (ER) where a signal peptidase removes the signal peptide [88,89]. The proteins are then glycosylated and further processed to ensure proper protein folding. Once proteins are properly folded and audited by the endoplasmic reticulum (ER) fidelity machinery, the proteins are transported to the Golgi where oligosaccharide chains are modified by addition of complex sugars. The addition of the mannose-6-phosphate (M6P) marker to the protein takes place through stepwise enzymatic reactions, and is later recognized by the mannose-6-phosphate receptor (M6Pr), on the trans-Golgi for export in clathrin-coated vesicles and fusion with early and late endosomes. The low pH of the endosomes initiates dissociation of the lysosomal hydrolase from the mannose-6-phosphate receptors (M6Pr). The hydrolase is subsequently delivered to the lysosome and the mannose-6-phosphate receptors (M6Pr) are recycled back to the Golgi for another round of protein transport.

Adding the mannose-6-phosphate requires N-acetylglucosamine phosphotransferase activity [90-92]. The mannose-6-phosphate receptors, 300 kDa CI-M6Pr and 46 kDa CD-M6Pr, are required for recognizing mannose-6-phosphate-containing lysosomal proteins [93]. With deficiencies of these genes, as in human mucolipidoses II (also called I-cell disease) patients, or in double knockout of both of these mannose-6-phosphate receptors, a portion of the lysosomal proteins do reach the lysosome, indicating the existence of mannose-6-phosphate receptor-independent trafficking pathways. Lysosomal integral membrane protein-2 (LIMP-2) [94], and sortilin-like receptor 1 (SORL1) [95-98], a multi-ligand type-1 receptor with similarity to the yeast carboxypeptidase Y sorting receptor Vps10 protein, are examples of proteins involved in such mannose-6-phosphate receptor-independent pathways. Deficiencies of these targeting proteins are implicated in Gaucher disease [99,100], myoclonus epilepsy [94,101] and Alzheimer's diseases [102].

The lysosome contains more than 50 acid hydrolases. They are responsible for breaking down the sugar, lipid, glycolipids, glycosaminoglycans, nucleic acids and proteins. These enzymes work together to contribute to the total catabolic function of the lysosome. In the endosomes and lysosomes, precursor hydrolases undergo further proteolytic cleavage to become fully active. The cleavage of proenzymes into active forms occurring within the greater lysosomal systems prevents premature activation of hydrolases outside of the lysosomes. Endogenous inhibitors of some of the hydrolases also exist to facilitate the compartmentalized activity of the lysosomal enzymes [103-106]. Intracellular metabolites may be upregulated in response to stress to modulate lysosomal enzyme activities [107]. Lysosomal membranes maintain the acidic pH within the lumen of the lysosome, and play a key role in importing proteins from the cytosol, contacting and fusing with other vesicles such as late-endosomes and autophagosomes [108-110], and exporting degradation products back to the cytosol [111-113]. Defects in lysosomal enzymes and membranes can cause neurodegenerative diseases [5,114-119] (Figure 1).

Lysosomes are dynamic organelles controlling protein and organelle homeostasis. Autophagy-lysosomal pathway-mediated proteolysis has been thought to be a bulk mechanism for protein turnover, serving as a common endpoint for multiple vesicle-based trafficking systems [120]. Proteins destined to be degraded in lysosomes can be delivered to these organelles by macroautophagy or by chaperone-mediated-autophagy. Macroautophagy delivers damaged proteins and organelles to the lysosome via autophagosomes. Autophagosome formation is initiated by *de novo *synthesis of double membrane vesicles or budding from endoplasmic reticulum, Golgi or mitochondria in the cytoplasm. These vesicles encircle aged or damaged proteins and organelles and deliver their cargos to lysosomes for degradation. Macroautophagy is induced by starvation to perform bulk recycling of proteins and organelles. In addition, nucleophagy [121], ribophagy [122], reticulophagy [123], mitophagy [124,125], and pexophagy [126] regulate clearance of specific complexes and organelles. Chaperone-mediated-autophagy is initiated when chaperones bind to a consensus sequence in target proteins, and deliver them to the lysosomes via the chaperone-mediated-autophagy receptor, lysosomal-associated membrane protein 2a (LAMP-2a), which is a splice variant of LAMP-2 [127]. In the next section, we will discuss how lysosomal dysfunction contributes to neurodegenerative diseases.

### 2b. Dysfunction of lysosomes in Parkinson's disease

**Figure 2:**
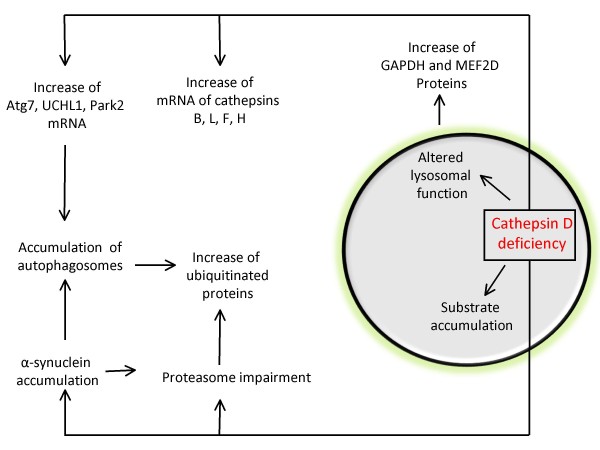
**Consequence of Cathepsin D deficiencies**. Cathepsin D is a major proteolytic enzyme in the lysosome. It cleaves α-synuclein in vitro and in cultured cells. Cathepsin D deficiency in human patients led to accumulation of lipofuscin and a prototype lysosomal storage disorder. Cathepsin D deficient worms, flies, mice, sheep and human patients exhibit increased α-synuclein accumulation and toxicity. Other associated cellular dysfunctions include: accumulation of autophagosomes, increased levels of Atg7, UCHL1, Park2, Cathepsin B, L, F, and H mRNAs, increased GAPDH and MEF2D proteins, impairment of proteasomal activities, and accumulation of ubiquitinated proteins. Overexpression of Cathepsin D in worms, flies, and mammalian cells has been shown to reduce α-synuclein aggregation and toxicity, suggesting a new approach to design future effective therapies.

The lysosomal system has been proposed to be a genetic "hotspot" for neurodegenerative diseases [5,6]. And different types of lysosomal deficiency lead to distinct disease phenotypes. Malfunction of specific enzymes in the lysosome have been associated with Parkinson's disease. Deficiencies at multiple steps of the pathway contribute to susceptibilities to disease pathogenesis (Figure 1). Inhibition of lysosomal activities may also have indirect adverse effects on other proteolytic pathways [128]. We will review i) known genetic lysosomal deficiencies in human patients that are associated with Parkinsonism or Parkinson's disease; ii) studies that examined lysosomal protein levels and activities in sporadic Parkinson's disease patients compared to normal controls; and iii) the most extensively studied lysosomal hydrolase, Cathepsin D, in relationship to protein aggregation and toxicity (Figure 2).

#### i) Parkinson's disease and Parkinsonisms associated with lysosomal mutations

Parkinsonism has been noted in lysosomal deficiencies such as adult forms of neuronal ceroid lipofuscinosis (ANCL) [129], Gaucher disease [130], and Kufor-Rakeb syndrome [131]. Three types of genetic lysosomal deficiencies have been found to generate Parkinsonism or Parkinson's disease pathologies, including deficiencies in lysosomal membrane function, breaking down of glycolipids and protein degradation. First, loss-of-function of a neuronal lysosomal membrane protein P-type ATPase, ATP13A2, underlies an autosomal recessive form of early-onset Parkinsonism with pyramidal degeneration and dementia (PARK9, Kufor-Rakeb syndrome) [131]. The mutation results in its mis-targeting and endoplasmic reticulum retention. Deficient ATP13A2 may lead to decreased lysosomal acidification and consequent deficient function of the lysosomes.

Second, a relatively large group of deficiencies in sugar or lipid breakdown enzymes are implicated in Parkinsonism and Parkinson's disease pathologies. Genome-wide association studies have suggested that mutations in glucocerebrosidase are an important risk factor for Parkinson's and other Lewy body disease [132,133]. Deficiencies in glycolipids degradation enzymes, as occurring in Gaucher disease [134], Niemann-Pick disease [135], GM2 gangliosidosis, Tay-Sachs, Sandhoff disease, metachromatic leukodystrophy, and beta-galactosialidosis [136], result in α-synuclein aggregation in both neurons and glia.

Interestingly, so far out of ~10 genes known that cause lysosomal storage disease neuronal ceroid lipofuscinosis (NCL), only tripeptidyl peptidase I and Cathepsin D are proteases. Parkinsonism has been observed in lysosomal tripeptidyl peptidase I deficient patients [137]. Cathepsin D homozygous inactivation in humans causes congenital neuronal ceroid lipofuscinosis (NCL) with postnatal respiratory insufficiency, status epilepticus, and death within hours to weeks after birth [138]. One patient with significant loss of Cathepsin D enzymatic function, due to compound heterozygous missense mutations, developed childhood motor and visual disturbances, cerebral and cerebellar atrophy, and progressive psychomotor disability [139]. In human patients with neuronal ceroid lipofuscinosis (NCL) due to a Cathepsin D deficiency, the brains were extremely atrophic with massive neuronal loss throughout the cortex, accompanied with intense α-synuclein staining [140]. Intense α-synuclein immunostaining also exists in the thalamus and basal ganglia [140]. Aggregates with various sizes localized to both proximal axons and the cell soma [140]. Aggregates in the cerebellum were localized to the granular cell layer and deep white matter [140]. Besides Parkinsonism and α-synucleinopathy, most of the neuronal ceroid lipofusinosis (NCL) patients' brains exhibit morphologically and biochemically deficient mitochondria [141,142].

#### ii) Expression levels of lysosomal proteins in Parkinson's disease

Aging brains exhibit reduced lysosomal function [6]. Increased accumulation of macroautophagic vesicles has been observed in postmortem Alzheimer's and Parkinson's disease patient brains compared to normal controls, consistent with either overproduction of macroautophagic vesicles or a deficit in macroautophagolysosomal clearance [143-146]. Recent studies demonstrated that macroautophagy is efficient in neurons and lysosomal activities are rate-limiting in clearance of protein aggregates [84]. While Loss-of-function of a neuronal lysosomal P-type ATPase, ATP13A2, underlies an autosomal recessive form of early-onset Parkinsonism, ATP13A2 mRNA expression is upregulated in sporadic Parkinson's disease [131]. Interestingly, Cathepsin D protein is upregulated in affected neurons in postmortem brains of patients with Alzheimer's disease; and is accompanied by autophagic vesicle accumulation as identified by immuno-electron microscopy [144,145]. These observations raise the possibility that the upregulation of lysosomal proteins serve as a compensatory mechanism for other lysosomal defects or in response to protein deposition pathology in sporadic Parkinson's disease and Alzheimer's disease [144,147-149]. Alternatively, the accumulation of Cathepsin D protein is a consequence of defects in its trafficking and its own degradation due to other lysosomal defects.

Not many publications directly investigated expression of lysosomal proteins in Parkinson's disease brains. One recent study has found that lysosomal protease Cathepsin D, Lysosome Associated Membrane Protein 1 (LAMP1), and Heat Shock Protein 73 (HSP73) immunoreactivities are significantly decreased (each protein to about 50%) in Parkinson's disease substantia nigra neurons. The down regulation is more severe in neurons that contained α-synuclein inclusions [150]. The accumulation of α-synuclein could be a result of impaired clearance by the lysosome via decrease in chaperone-mediated-autophagy or specific decrease in Cathepsin D protein levels.

Overexpression of mutant human α-synuclein in cultured cells induces lysosomal dysfunction, accumulation of autophagosomes and many of these accumulated autophagosomes contain engulfed mitochondria [151]. Furthermore, Parkinson's disease causing A53T, A30P mutant α-synuclein, or S129 site phosphorylated α-synuclein blocks chaperone-mediated-autophagic activity [127,152]. Overexpression of mutant human α-synuclein in the rat substantia nigra using viral vectors leads to reduction of Lysosome Associated Membrane Protein 1 (LAMP1), and Heat Shock Protein 73 (HSP73) levels in neurons with α-synuclein immunoreactive inclusions. Interestingly, mutant α-synuclein expressing rat neurons without inclusions exhibit higher Cathepsin D immunoreactivity compared to controls. One interpretation is that an early and effective enhancement of Cathepsin D expression in a subset of neurons delays or attenuates the accumulation of α-synuclein. The subset of neurons unable to enhance Cathepsin D activity consequently suffers from a vicious cycle of α-synuclein accumulation and further reduction of Cathepsin D [150].

#### iii) Cathepsin D deficiency in vitro and in animal models

Cathepsin D is the principal lysosomal aspartate protease and a main endopeptidase. Cathepsin D is synthesized as a prepropeptide of 51 kDa, once the signal peptide is cleaved upon Cathepsin D insertion into the endoplasmic reticulum the resultant propeptide is 49 kDa. It has been shown that Cathepsin D is delivered to the lysosome via the mannose-6-phosphate receptor (M6Pr)-dependent pathway, however it can also be delivered to the lysosome in a mannose-6-phosphate receptor (M6Pr)-independent manner via binding to sortilin (67). The Cathepsin D zymogen is then activated in the acidic lysosomal environment [153]. Its yeast homolog, PEP4, plays a critical role in maturation of other vacuolar proteases, total cellular protein turnover under normal and nutrient-deprived conditions, and total cellular protein turnover in response to oxidative stress [154-157].

Cathepsin D knockout (*ctsd*-/-) mice die at approximately postnatal day 26 (P26), secondary to a combination of nervous system and systemic abnormalities, including both accumulation of autophagic vacuoles at as early as postnatal day 0 (P0). These *ctsd*-/-mice exhibit seizures, unsteady posture, and smaller brains. No overt loss of substantia nigra dopaminergic neurons was observed, likely due to the young age [158-162]. Significantly, α-synuclein accumulation in neuronal cell bodies has been found in P25 *ctsd*-/- but not wildtype cortex [163]. In keeping with these observations, elevated levels of high molecular weight but not monomeric α-synuclein, and high molecular weight ubiquitinated proteins have been found in extracts from the cortex of *ctsd*-/- mice but not wildtype mice; these findings are reminiscent of what is seen in Lewy body diseases. The cytoplasmic microtubule-associated protein, tau, or the synaptic protein, synaptophysin, did not accumulate in *ctsd*-/- cortex compared to wildtype cortex [163], suggesting that Cathepsin D deficiency at P25 does not have a general effect on the accumulation of all cytoplasmic and synaptic proteins.

In contrast to the brains of human lipidoses patients [136], where α-synuclein aggregates are found in both neurons and glia and co-localize with lipids, in *ctsd*-/- brains α-synuclein accumulations do not co-localize with autofluorescent lipofuscin. Furthermore, α-synuclein accumulations in *ctsd*-/- brains are present in neurons but not in astrocytes. In a relatively small fraction of neurons (~5%), prominent accumulations of α-synuclein immunoreactivity co-localize with intense ubiquitin staining, consistent with the observation that a small fraction of α-synuclein in Lewy bodies is ubiquitinated [164,165]. Along similar lines of increased autophagy in *ctsd*-/- mice, an increase in mRNA encoding autophagy related proteins Atg7, Parkinson's disease genes UCHL1 and Park2, as well as Cathepsins B, L, F, and H has been observed. In addition, substrates for chaperone-mediated-autophagy, GAPDH and MEF2D proteins, are increased in *ctsd*-/- mice, suggesting a reduction of chaperone-mediated-autophagy [163,166]. In a worm model, Cathepsin D reduction by siRNA exacerbates α-synuclein aggregation from transgenic expression of a human α-synuclein gene [163]. Figure 2 summarizes the effects of Cathepsin D deficiency on autophagy and α-synuclein accumulation. As one major lysosomal acid hydrolase, Cathepsin D deficiency likely contributed to the accumulation of lysosomal storage of lipofuscins in the Cathepsin D deficient brains. Although has not been directly tested, other aspects of lysosomal function, such as lysosomal membrane property, ability of the lysosomes to fuse with autophagosomes, and the efficiency of the lysosomes to export degradation products, may also be affected as an indirectly consequence of Cathepsin D deficiency.

In a parallel study, Cullen et al investigated α-synuclein immunoreactivity in a number of species deficient in Cathepsin D [140]. Detailed immunohistochemical studies have found that in Cathepsin D-deficient mice, α-synuclein accumulation occurs in neurons from the deep cortical laminae, superior colliculus, subiculum, thalamus, deep cerebellar nuclei, and the white matter tracts. In Cathepsin D-deficient sheep, widespread axonal swelling in deep white matter tracts, and isolated α-synuclein aggregates in the thalamus have been found. In transgenic flies that express human α-synuclein, Cathepsin D deficiency exacerbated retinal degeneration.

Cathepsin D knockout mice have the most severe phenotypes of all cathepsin knockouts. In mice, Cathepsin F deficiency leads to phenotypes resembling late onset neuronal ceroid lipofuscinosis (NCL) [167]. Cathepsin B and L appear to be able to compensate for each other in the nervous system to a certain extent. In support of this conclusion, mice with deficiencies in both Cathepsin B and L genes exhibit phenotypes resembling mice with single deficiency of Cathepsin D [158].

## 3. Upregulation of lysosomal genes as a potential therapeutic approach against Parkinson's disease

Enhancing autophagy can reduce protein aggregation in a number of cellular models of neurodegenerative diseases [168-174]. The effects of enhancing macroautophagy at distinct steps can be additive in enhancing protein degradation [175]. There are two limitations to this approach. First, because both macroautophagy and chaperone-mediated-autophagy are dependent on intact lysosomes, it is essential that lysosomal activities are preserved to be effective in clearing potentially neurotoxic proteins. Second, enhancing overall macroautophagy may be detrimental because of the risk of reducing normal proteins. In comparison, enhancing lysosomal function may have the advantage of increasing the efficiency of turnover of autophagosome-sequestered damaged proteins and organelles, without sacrificing selectivity for degradation of toxic or dysfunctional cellular materials. Studying upregulation of lysosomal genes as a potential therapeutic approach against Parkinson's disease has just recently come to play, and is of limited scale. Thus, we focus this part of our review on the study of Cathepsin D.

### 3a. Reduction of α-synuclein by Cathepsin D

Mammalian Cathepsin D recognizes α-synuclein at Y125 *in vitro *[176]. Nitration and phosphorylation of α-synuclein at Y125 have been observed in cultured cells although whether these changes are required for initiation of α-synucleinopathy is unclear [177-181].

Studies using Cathepsin D inhibitors and siRNA demonstrated that lysosomal Cathepsin D is the most active in degradation of α-synuclein [178]. In an aggregation assay in H4 neuroglioma cells, co-over-expression of synphilin and an α-synuclein-green fluorescent protein (GFP) fusion protein (α-synuclein-GFP) forms visible aggregates in transfected H4 cells [182]. Transfection of Cathepsin D gene (*CTSD*) together with α-synuclein-GFP and synphilin reduces α-synuclein aggregates [163]. Immunocytochemistry studies indicated that the effects of Cathepsin D on reduction of α-synuclein aggregation are likely due to reduction of α-synuclein rather than secondary to a reduction in synphilin levels [163]. By transfecting Cathepsin D gene (*CTSD*) in a rodent cell line of mesencephalic origin MES23.5, Cullen et al have found that Cathepsin D is effective in reducing both the wildtype and 7 different mutant forms of α-synuclein: the three Parkinson's disease-linked mutant A30P, A53T and E46K, the serine 129 mutant S129A and S129D, as well as the D98A and Q99A mutant abolishing chaperone-mediated-autophagy [140].

Excessive α-synuclein induces neuron death in cell cultures, and in a variety of genetic and viral delivery-based animal models [33,34,183,184]. Overexpression of α-synuclein-GFP induced robust cell death in SH-SY5Y cells. Co-transfection of human Cathepsin D provided significant protection against α-synuclein-GFP overexpression-induced cell death. Cathepsin D has a known specificity for hydrophobic residues [176]. Although α-synuclein contains many putative Cathepsin D cleavage sites, Y125 is the primary site of cleavage [176]. We found that Cathepsin D is also protective against cell death induced by mutant α-synuclein (A30P and A53T) in SH-SY5Y cells. In contrast, mutating α-synuclein at Y125 [176] results in an α-synuclein mutant that induces cell death and resists neuroprotection by elevated Cathepsin D. Furthermore, Cathepsin D is ineffective at attenuating chloroquine-or staurosporine-induced cell death. In *C. elegans*, overexpression of wildtype Cathepsin D, but not Cathepsin D enzymatic mutants, Cathepsin B or Cathepsin L, protects against α-synuclein toxicity, indicating a conserved mechanism of Cathepsin D in cytoprotection [163].

Other lysosomal proteins have been shown to protect against α-synuclein toxicity include ATP13A2 [185], a protein that may be involved in maintaining lysosomal acidic environment. As mentioned above, transcription factor EB (TFEB) elicits a coordinated upregulation of genes involved in lysosomal activities and transporting substrates to the lysosomes [87]. TFEB overexpression has been shown to reduce mutant Huntingtin load in a rat striatal cell line, HD43 cells [87]. These findings will surely inspire a series of future studies to determine whether these and other proteins involved in lysosomal function can provide novel therapies against Parkinson's and other neurodegenerative diseases.

## Conclusions and perspectives

Neurons are non-dividing and rarely replenishing cells that cannot distribute accumulated by-products through mitosis, thus needing a diligent cleaning system for maintenance of cell health. Lysosomes are the major sites for degrading aged proteins and organelles. Thus lysosomes are essential for cells to maintain proper protein and organelle quantity and quality, and of primary importance in the context of avoiding neurodegenerative diseases, including Parkinson's disease. As such, many genetic mutations with dysfunctional lysosomes lead to neurological diseases, including Parkinsonism and Parkinson's pathogenesis. Fortunately, the finding that lysosomal activities are rate-limiting in degradation of aged and damaged proteins and organelles provides an opportunity to examine the potential of upregulating lysosomal activities to help prevent, attenuate or even reverse the manifestation of Parkinson's disease. The observation that lysosomal activities can be regulated at the level of transcription, trafficking, processing and activation offers a number of potential approaches to develop therapeutic strategies for neurodegenerative diseases.

## Abbreviations

(**AAV**): adeno-associated virus; (**CD**): Cathepsin D; (**ER**): endoplasmic reticulum; (**LAMP-2a**): lysosomal-associated membrane protein 2a; (**LIMP-2**): lysosomal integral membrane protein-2; (**M6Pr**): mannose-6phosphate receptor; (**mitophagy**): autophagy of mitochondria; (**MPP+**): 1-methyl-4-phenylpyridinium; (**MPTP**): 1-methyl 4-phenyl 1,2,3,6-tetrahydropyridine; (**PD**): Parkinson's disease; (**TFEB**): transcription factor EB.

## Competing interests

The authors declare that they have no competing interests.

## Authors' contributions

LS and JZ wrote the review. All authors have read and approved the final manuscript.
